# Types, characteristics and anatomic location of physical signs in elder abuse: a systematic review: Awareness and recognition of injury patterns

**DOI:** 10.1007/s41999-021-00550-z

**Published:** 2021-09-13

**Authors:** Miriam E. van Houten, Lilian C. M. Vloet, Thomas Pelgrim, Udo J. L. Reijnders, Sivera A. A. Berben

**Affiliations:** 1 Department of Geriatrics, Radboud University Medical Centre, PO Box 9101, 6500 HB Nijmegen, The Netherlands; 2 Research Department of Emergency and Critical Care, Knowledge Centre of Sustainable Healthcare, School of Health Studies, HAN University of Applied Sciences, PO Box 6960, 6503 GL Nijmegen, The Netherlands; 3 Trompetter and Partners Social Medical Expertise, Utrechtseweg 75, 3702 AA Zeist, The Netherlands; 4 Radboud Institute for Health Sciences IQ Healthcare, Radboud University Medical Centre, P.O. Box 9101, 6500 HB Nijmegen, The Netherlands; 5 School of Health Studies, Knowledge Centre of Sustainable Healthcare, HAN University of Applied Sciences, PO Box 6960, 6503 GL Nijmegen, The Netherlands; 6 Department of Forensic Medicine, Amsterdam Public Health Service, PO Box 2200, 1000 CE Amsterdam, The Netherlands

**Keywords:** Bruises, Physical signs, Elder abuse, Distribution, Forensics

## Abstract

**Purpose:**

Elder abuse is a worldwide problem with serious consequences for individuals and society. The recognition of elder abuse is complex due to a lack of awareness and knowledge. In this systematic review, types, characteristics and anatomic location of physical signs in elder abuse were identified.

**Methods:**

Databases of MEDLINE, COCHRANE, EMBASE and CINAHL were searched. The publication dates ranged from March 2005 to July 2020. In addition to the electronic searches, the reference lists and citing of included articles were hand-searched to identify additional relevant studies. The quality of descriptive and mixed-methods studies was assessed.

**Results:**

The most commonly described physical signs in elder abuse were bruises. The characteristics of physical signs can be categorized into size, shape and distribution. Physical signs were anatomically predominantly located on the head, face/maxillofacial area (including eyes, ears and dental area), neck, upper extremities and torso (especially posterior). Physical signs related to sexual elder abuse were mostly located in the genital and perianal area and often accompanied by a significant amount of injury to non-genital parts of the body, especially the area of the head, arms and medial aspect of the thigh.

**Conclusions:**

Most common types, characteristics and anatomic location of physical signs in elder abuse were identified. To enhance (early) detection of physical signs in elder abuse, it is necessary to invest in (more) in-depth education and to include expertise from a forensic physician or forensic nurse in multidisciplinary team consultations.

**Supplementary Information:**

The online version contains supplementary material available at 10.1007/s41999-021-00550-z.

## Key summary points

**Aim:**

Identify types, characteristics and anatomic location of physical signs in elder abuse.

**Findings:**

Physical signs in elder abuse are most common bruises and anatomically predominantly located on the head, face/maxillofacial area, neck, upper extremities and torso.

**Message:**

Increase knowledge on physical signs in elder abuse so as to enhance timely detection and intervention.

**Supplementary Information:**

The online version contains supplementary material available at 10.1007/s41999-021-00550-z.

## Background

Elder abuse is a worldwide problem with serious consequences for individuals and society, due to increased morbidity, mortality and use of healthcare resources, especially emergency services [1–3]. The definition of elder abuse is formulated by the World Health Organization (2017) as “a single or repeated act, or lack of appropriate action, occurring within any relationship where there is an expectation of trust, which causes harm or distress to an older person”. There are various forms of elder abuse: financial, physical, psychological and sexual abuse. Elder abuse can also be the result of intentional or unintentional neglect. Based on available evidence it is estimated that 15.7% of people of 60 years and older worldwide are subjected to abuse [4]. This prevalence rate is likely to be an underestimate, as many cases of elder abuse are not reported. Furthermore, studies on prevalence rates in elder abuse often show heterogeneity due to regional and cultural differences between countries or varying definitions of elder abuse (for example with regard to age cutoff point) used. In the Netherlands, 1 in 20 people aged 65 years and over living at home experience elder abuse at some point in their lives, and 1 in 50 people aged 65 years and over living at home experience elder abuse on an annual basis [5].

There is complexity in the recognition of elder abuse. The level of awareness and knowledge on elder abuse in healthcare professionals is still poor and there is a strong need for education and specific training on recognition [6, 7]. On the other hand, older persons will not always report circumstances of abuse because of cognitive and/or speech impairment [8]. But even if they are able, they will not always report being a victim of elder abuse because of fear from repercussions from the abuser, issues of shame or loyalty [8, 9]. Interactions with healthcare professionals, such as physicians and nurses in the hospital setting, present crucial opportunities to recognize elder abuse and to intervene or to refer to the appropriate authorities [8]. Also signs of elder abuse are often detected in acute situations such as admittance to the ED (emergency department). Professionals in the ED may be the first healthcare professionals to have contact with the older persons. A study from Dong et al. [10] showed that older persons who experienced two or more types of elder abuse also had significantly higher rates of ED use. Also, they were less likely to hide signs of elder abuse in acute situations such as admittance to the ED.

Different types of elder abuse, such as physical abuse, sexual abuse and neglect, can cause physical injuries. The detection and recognition of physical signs related to elder abuse may be complicated because it is not always easy to discriminate from signs of underlying diseases. For example, age-related changes or certain medication can make the skin more vulnerable to injury, which makes it difficult to assess whether skin bruising is either of an accidental or of a non-accidental nature. Furthermore, there are no known pathognomonic physical signs of elder abuse described, unlike in certain cases of child abuse [11–13].

In this systematic review, we aimed to identify the types (e.g., bruises), characteristics (e.g., size, shape and distribution) and anatomic location of physical signs in elder abuse to increase the awareness and recognition on injury (patterns) by clinical geriatricians and other healthcare professionals.

## Methods

### Design

A systematic review of the literature was performed according to the steps of the Cochrane Handbook for Systematic Reviews of Interventions [14], and reported in concordance with the Preferred Reporting Items for Systematic reviews and Meta-Analyses (PRISMA) statement [15].

### Search strategy

The databases of MEDLINE, COCHRANE, EMBASE and CINAHL were searched. The publication dates ranged from March 2005 to July 2020. In addition to the electronic searches, the reference lists and citing of included articles were hand-searched to identify additional relevant studies. The search strategy was partly based on available MeSH terms from the search strategy protocol of the *Cochrane review on Interventions for preventing abuse in the elderly* [16]. Furthermore the (modified) search strategy protocol from the chapter on the recognition of physical signs related to elder abuse from the Dutch guideline on suspected elder abuse (NVKG 2018) was used [17]. The full search strategy per database is provided in Supplementary Information Text 1.

### Study selection procedure

**Fig. 1:**
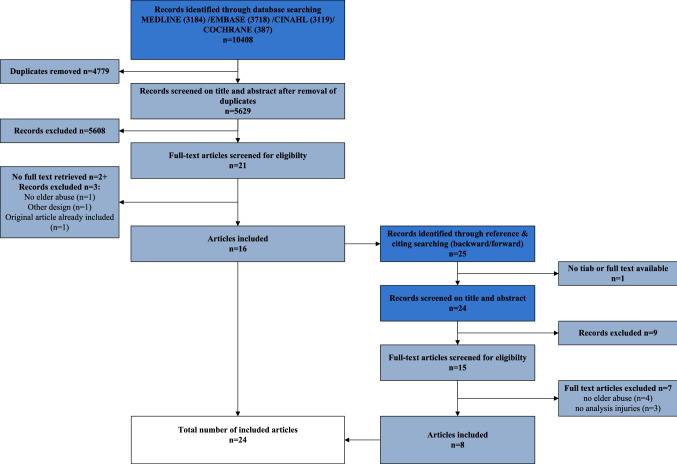
Study selection process

All types of reviews, quantitative and qualitative study designs were included, with the limitation of studies published in the Dutch, German, French and English language. The inclusion criteria were: studies containing a description of types of physical signs (related to elder abuse) with regard to their characteristics and/or anatomic location of physical signs. Excluded were: conference proceedings, editorials, or other personal communications and studies that focused on the prevalence of elder abuse, or legislation and education in elder abuse not related to physical injuries. Furthermore, studies on suicide, homicide, histological examination, use of restraints from a professional perspective or self-neglect of older persons were excluded. All articles were screened on title and abstract by two independent reviewers (SB, MVH). In case of doubt, a third reviewer (LV) was asked to make a final decision. In addition, reference lists and citing of included articles were screened (SB, MVH) and potentially relevant new publications were screened in a similar way (see Fig. 1 for study selection process).

### Quality assessment

To assess the quality of the descriptive studies, we used the 14-criteria quantitative tool from Kmet et al. [18]. We deleted three criteria from the tool (criteria five, six, and seven) regarding experimental research. The quality assessment was performed by two independent researchers (SB, MVH). To assess the quality of the mixed methods studies, a multimethod validated appraisal tool (MMAT version 2018) was used [19]. The MMAT is the only tool that includes specific criteria for mixed methods studies. With its five different sets of criteria, the MMAT uses a combination of individual component and mixed methods approaches. Any disagreements in criteria ratings between reviewers were discussed until a consensus was reached. No quality assessment was performed for the narrative reviews and case report studies. Instruments for the quality assessment of narrative reviews have been developed, but were not used in this systematic review because the results of narrative reviews were mostly based on the primary studies that were already included in this systematic review. The case report studies were mainly descriptions of individual patients where a quality assessment was not deemed to be of added value.

### Data extraction

Data were extracted by two independent researchers (SB, MVH). Outcomes extracted were:

1. Types of physical signs in elder abuse.
2. Characteristics of physical signs in elder abuse.
3. Anatomic location of physical signs in elder abuse.

### Data synthesis and presentation

Due to the paucity of original studies, we analyzed and synthesized all studies, by scrutinizing and categorizing data. The case report data from the mixed methods studies were considered as original data and were therefore analyzed as case report studies. The primary outcomes were based on descriptive studies. Additionally, information from other study designs was added. First, studies were categorized according to their design or publication form. Second, three themes based on the taxonomy for visible intentional and unintentional acute injuries by Rosen et al. [20] were modified for this study and used for classification of the data extraction: (1) Types of physical signs. (2) Characteristics of physical signs. (3) Anatomic location of physical signs.

The following anatomic locations were chosen to categorize the physical signs:

1. Skull/brain/maxillofacial/dental/neck.
2. Chest/abdomen/back.
3. Extremities (upper/lower).
4. Pelvis/gluteal.
5. Extragenital in sexual elder abuse.
6. Miscellaneous.

## Results

### Review statistics

The initial search identified 5629 unique records, after the selection procedure 24 studies were included (see Fig. 1).

### Study characteristics

The design of the included studies concerned eight descriptive studies [13, 21–27], three case studies [28–30], five narrative reviews [11, 31–34], six mixed methods studies [12, 35–39] and two books [40, 41]. See Tables 1, 2, 3 and 4 for characteristics studies.

**Table 1:** Characteristics descriptive and case report studies (*n* = 11)

1st author Year Country	Design	Aim	Methods/data resources	Setting	Patients (*n*) age
Abath 2010 Brazil	Retrospective document study	To describe the profile of physical abuse among older people	Forensic examination reports (*n* = 1.027) of physical abuse patients (2004—2007) were analyzed for the following variables: -Characteristics of the event, victim and aggressor -The consequences of the physical abuse	Institute of Forensic Medicine	Patients (*n* = 1.027) who were victims of physical abuse and underwent forensic examination Sex: 59.2% male. The most common age bracket was 60–69 years. The proportion of cases in this age bracket was 12.3 times that of the 80-and-older group
Burgess 2005 USA	Retrospective document study	To describe essential forensic markers unique to older adult victims of sexual abuse	Patient records of sexual abuse cases (year not described) submitted by experts were analyzed for the following variables: -Victim/offender characteristics and patterns of behavior -Mechanisms/patterns of injury -Forensic data -Criminal justice process and outcomes -Comparison community-based victims/nursing home victims	Home setting (53%) Inpatient setting (44%) Other place (3%)	Patients (*n* = 125) who were victims of sexual abuse Sex: 100% female Mean age: 78.48 ± ? Years (min 60; max 98)
Cham 2000 Singapore	Retrospective document study	To describe the frequency and characteristics of elder abuse	Elder abuse cases were selected from elderly patients (*n* = 62,826) visiting the emergency department (ED) (1994–1997) From the elder abuse cases the following variables were analyzed: -Characteristics of the victims/perpetrators, -Characteristics of injuries -Event circumstances -Involvement police/social workers	Emergency department	Patients (*n* = 17) who were victims of elder abuse Sex: 82.3% female Mean age: 74.6 ± ?years
Kavak 2019 Turkey	Retrospective document study	To describe the radiologic imaging characteristics of trauma-related lesions in elder abuse patients	Patient records and radiological images of patients (*n* = 92) visiting the emergency department (ED) with fracture(s) (2013–2018) who were established to be abused (*n* = 92) were analyzed for the following variables: -Age, gender -Reason for adm. to the hospital -Presence/absence comorbid disease(s) -Bone fracture location and number -Characteristics of the fracture(s) -Presence/absence soft-tissue damage or old fracture(s) -Mortality	Emergency department	Patients (*n* = 92) with a diagnosis of elder abuse and a minimum one fracture in at least one bone in radiologic imaging Mean age 73.2 ± 5.87 years
Rosen 2016 USA	Retrospective document study	To describe patterns and circumstances surrounding elder abuse-related and potentially elder abuse-related injuries in older adult ED patients	Elder Protective Service (EPS) physical abuse cases (*n* = 111) (between 1985 and 1992) were matched to patient records of emergency department (ED) visits during a 5-year period (1981–1994) before or after the date of the verified physical abuse and each ED visit was evaluated The following variables were analyzed: -Probability of injuries related to elder abuse -Characteristics victims/perpetrators -Household items used to inflict injuries -Injury patterns -Presence of suspicious circumstances surrounding the ED visit or suspicious injury patterns	Emergency department	ED patients (*n* = 26) with abuse-related injuries, 81% female, age not described ED patients (*n* = 57) with injuries not identified as due to abuse, 81% female, age not described
Rosen 2020 USA	Prospective study with matched case control group	To describe differences between injury patterns associated with physical elder abuse and those associated with unintentional falls	Elder abuse cases (successfully prosecuted) from the King’s County District Attorney’s Office (*n* = 100) were retrospectively examined for the following variables: -The injuries -The victim -The abuser -The circumstances surrounding the physical abuse incident and its detection Patients aged 60 years or older who presented to the ED after an unintentional fall (*n* = 578) were prospectively enrolled (2014–2018) and were examined for the following variables: -Demographics -Health -Functional status -Circumstances surrounding the fall injury -The characteristics of the injuries	Elder abuse cases: not mentioned Prospective group: emergency department	Patients (*n* = 78) with successfully prosecuted elder abuse cases with visible injuries (*n* = 264) resulting from the abuse Matched patients (*n* = 78) with visible injuries (*n* = 217) after an unintentional fall case patients and controls had a mean age of 71 years ± 9 years Sex: 73% female
Wiglesworth 2009 USA	Prospective observational study	To describe bruising as a marker of physical elder abuse	Patients from APS (adult protective services) (*n* = 407) (2006–2008) who were physically abused were approached to participate in the study within 30 days of the abuse incident The following variables were measured: -Age, sex, ethnicity, race -functional status -Medical conditions -Cognitive status -History of falls -Bruise size and location -Recall of cause -Responses to Revised Conflicts Tactics Scale and Elder Abuse Inventory A baseline comparison group from a prospective documentation study of older persons with accidental bruising (not related to elder abuse) was added to the study	Home or inpatient setting	Patients from APS (*n* = 67) who were victims of physical abuse, *n* = 48 had bruises Age: 77.5 ± 8.1 Sex: 32.2% male Patients (*n* = 68) with accidental bruising Age: 88.5 ± 5.7 Sex: 27.9% male
Ziminski 2013 USA	Secondary data analysis Wigglesworth 2009	To describe mechanisms of injury in association with characteristics of bruising in physical elder abuse	Data from patients from adult protective services (APS) (*n* = 67) (2006–2008) were included and evaluated The following variables were collected: -Demographics -Number of falls -Medical history/diagnoses and medications Furthermore characteristics of bruises were analyzed and CTS2 (Revised Conflict Tactics Scale) items were used to represent the mechanisms of injury and the association with bruising locations	Home or inpatient setting	Patients from APS (*n* = 67) who were victims of physical abuse, *n* = 48 had bruises Age: 77.5 ± 8.1 Sex: 32.2% Male
Speck 2014 USA	Case report series	To describe cases of (possible) sexual abuse	Nine cases are described to show: -Perpetrator schemes, -Traumatic reactions from victims -Interventions to care for the victims	Institutional setting Domestic community setting	Patients (*n* = 9) Mean age ± SD (sex): Case 1: 84 (female) = no elder abuse, urethral trauma after traumatic removal during reactions to paranoid hallucinations Case 2: 65 (female) = no elder abuse, was sexual assault by stranger and no formal caretaker Case 3: 68 (male) Case 4: 70 (female) = no elder abuse, sexual assault by stranger Case 5: 88 (female) Case 6: 65 (female) Case 7: 78 (female) = no elder abuse, consenting sexual activity Case 8: 72 (female) = no elder abuse, fungal infection and mental illness Case 9: 70 (male) = no elder abuse, sexual abuse by resident
Young 2014 USA	Case report study	To describe cases and presenting symptoms of physical elder abuse	Four cases are described to show variety of symptoms in physical elder abuse	Institutional setting Domestic community setting	Patients (*n* = 4) Mean age ± SD (sex): Case 1: 90 year (female) Case 2: 75 year (male) Case 3: 91 year (female) Case 4: 82 year (female)
Wong 2017 USA	Case report study	To describe the imaging characteristics in cases of elder abuse	Two cases are described to show radiographic findings in elder abuse	Primary care setting Emergency department	Patients (*n* = 2) Mean age ± SD (sex): Case 1: 98 year (female) Case 2: 90 Year (female)

**Table 2:** Characteristics review studies (*n* = 5)

1st author Year Country	Design	Aim	Databases	Search strategy	Inclusion criteria	Included articles
Brown 2004 Country not described	Review	Aim not described, overview regarding position of nurse practioners in the inter-vention and detection of elder abuse	Not described	Not described	Not described	Not described
Clarysse 2018 Belgium	Review	To describe visible injuries of physical abuse, sexual abuse, and neglect	Not described	Not described	Not described	Not described
Collins 2006 USA	Review	To describe current medical and psychological understanding of elder maltreatment	Not described	Not described	Not described	Not described
Murphy 2013 Canada	Review	To describe risk factors and signs of elder abuse	1. PubMed 2. CINAHL 3. EMBASE 4. TRIP	Databases were searched from 1975 to March 2012 using the following words and phrases: “physical elder abuse”, “older adult abuse”, “elder mistreatment”, “geriatric abuse”, “geriatric trauma”, and “nonaccidental geriatric injury” in the titles of articles. Additional papers identified through reference lists Exclusion criteria: articles non-pertinent or duplication on screening of abstracts To summarize all the findings from these studies, physical injuries were classified according to anatomic location	Description of the types and distribution of physical injuries in elder abuse	9 articles: 1 case report 4 case series 2 case–control studies 2 cross-sectional descriptive studies
Pearsall 2005 USA	Review	To describe and analyze forensic biomarkers for elder abuse	Not described	Not described	Not described	Not described

**Table 3:** Characteristics: mixed methods studies (*n* = 6)

1st author Year Country	Design mixed methods	Design	Aim	Databases	Search strategy	Inclusion criteria	Included articles
Chang 2013 USA	Review and case reports	Review	To describe cutaneous manifestations of elder abuse	Not described	Not described	Not described	Not described
Danesh 2015 USA	Review and case reports	Review	To describe role of dermatologists in detecting elder abuse and neglect	Not described	Not described	Not described	Not described
Gibbs 2014 USA	Review and case reports	Review	To describe visible signs of physical abuse, sexual abuse, and neglect	Not described	Not described	Not described	Not described
Rohringer 2020 Canada	Review and case reports	Review	To identify injury findings specific to elder abuse	1. MEDLINE 2. Reference lists of selected articles were also explored	Databases were searched from 1995 to 2019 using the following search terms: Search terms included were: “radiological findings” or “radiographic findings” or “imaging” or “imaging findings” or “diagnostic imaging” or “medical imaging” or “CT” or “MRI” or “X-ray” and “elder abuse.” The reference lists of the selected articles were also explored	English-language articles relevant to the characterization of elder abuse	Not described
Palmer 2013 USA	Review and case reports	Review	To describe risk factors, signs, reporting requirements, and prevention of elder abuse	Not described	Not described	Not described	Not described
Russo 2019 Italy	Review and case reports	Review	The describe role of diagnostic imaging in the detection of lesions in domestic abuse in elderly patients and domestic abuse in women	Not described	Not described	Not described	Not described
Chang 2013 USA	Review and case reports	Case reports	To demonstrate cutaneous manifestations of elder abuse	Photo case reports	Emergency department and outpatient setting	Not described	Case reports (*n* = 3) Case 1: age/sex: not described Case 2: female, age not described Case 3: age/sex: not described other cases self-neglect(*n* = 1) or no elder abuse (*n* = 4) or no proven elder abuse (*n* = 2)
Danesh 2015 USA	Review and case reports	Case reports	To demonstrate visible physical signs of elder abuse	Photo case reports	Not described	Not described	Case reports (*n* = 4) Case 1: age/sex: not described Case 2: age/sex: not described Case 3: age/sex: not described Case 4: age/sex: not described Other cases: no elder abuse (*n* = 4)
Gibbs 2014 USA	Review and case reports	Case reports	To describe and demonstrate visible signs of physical abuse, sexual abuse, and neglect	Photo case reports Case report (narrative)	Photos: not described Narrative case report: community setting	Not described	Photo case reports (*n* = 23) of elder abuse (no age/sex) Narrative case report:(*n* = 1) male, no age
Rohringer 2020 Canada	Review and case reports	Case reports	To describe imaging findings in elder abuse	Case report	Not described	Not described	Patients (*n* = 2) Mean age ± SD (sex): Case 1: female, 63 Case 2: male, 70
Palmer 2013 USA	Review and case reports	Case reports	To demonstrate visible physical signs of elder abuse	Photo case reports	Not described	Not described	Photo case reports (*n* = 4) of elder abuse Case 1: age/sex: not described case 2: age: female, age not described Case 3: 70/sex not described Case 4: age/sex: not described other case no elder abuse (*n* = 1)
Russo 2019 Italy	Review and case reports	Case reports	To demonstrate diagnostic imaging of lesions in domestic abuse in elderly patients and domestic abuse in women	Photo case reports	Not described	Not described	Photo case reports of elder abuse (*n* = 3) Case 1: male, 72 Case 2: male, 71 Case 3: male, 76 Other case: no elder abuse

**Table 4:** Characteristics books (*n* = 2)

1st author Year Country	Design	Aim	Content
Baccino 2020 France	Book	Not described	The title of the chapter is “Imaging and Elderly abuse” Described are: 1. Background of elder abuse: definitions, epidemiology, signs and diagnosis 2. Particularities of imaging in elderly 3. Some imaging findings in elder abuse
Dyer 2002 USA	Book	Not described	The title of the chapter is "The clinical and Medical Forensics of Elder Abuse and Neglect". Described are several potential forensic markers of elder abuse and neglect

### Quality assessment

Most of the descriptive studies (*n* = 8) showed moderate to good quality [13, 21, 24, 26, 27].

Most of the mixed methods studies (*n* = 6) showed low quality [12, 35–37]. Despite the varying quality, all studies were included in our analysis. See Tables 5 and 6.

**Table 5:**
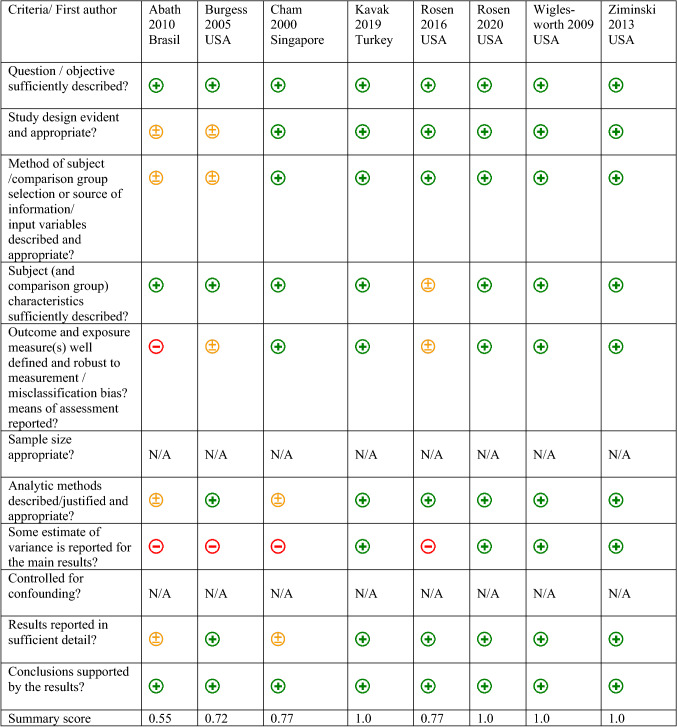
Quality of descriptive studies (*n* = 8)

Yes  Partial  No, N/A: not applicable; Total sum = (number of “yes” * 2) + (number of “partials” * 1); Total possible sum = 22 − (number of “N/A” * 2); Summary score: total sum/total possible sum; please note: 22 instead of 28 total possible sum because of only 11 items instead of 14 items

**Table 6:** Quality of mixed methods studies (*n* = 6)

Criteria/first author	Chang 2013 USA	Danesh 2015 USA	Gibbs 2014 USA	Rohringer 2020 Canada	Palmer 2013 USA	Russo 2019 Italy
Is there an adequate rationale for using a mixed methods design to address the research question?	Can’t tell	Can’t tell	Can’t tell	Yes	Can’t tell	Yes
Are the different components of the study effectively integrated to answer the research question?	Yes	Yes	Yes	Yes	Yes	Yes
Are the outputs of the integration of qualitative and quantitative components adequately interpreted?	Yes	Yes	Yes	Yes	Yes	Yes
Are divergences and inconsistencies between quantitative and qualitative results adequately addressed?	No	No	No	No	No	No
Do the different components of the study adhere to the quality criteria of each tradition of the methods involved?	No	No	No	Yes	No	No

### Outcomes: descriptive and case report studies (see Tables 7 and 8)

**Table 7:** Results descriptive and case report studies: types elder abuse and physical signs

1st author (Year) Country	Types: elder abuse	Types: physical signs
Descriptive studies		
Abath 2010 Brazil	1. PA	1. Burns
Burgess 2005 USA	1. SA	1. Abrasions 2. Bruises
Cham 2000 Singapore	1. PA	1. Blunt trauma 2. Bruises 2. Contusions 3. Dehydration 4. Fracture
Kavak 2019 Turkey	1. PA 2. N 3. PsA 4. FA	1. Fractures 2. Soft tissue lesions
Rosen 2016 USA	1. PA	1. Bruises 2. Fracture 3. Hematoma (subdural) 4. Laceration
Rosen 2020 USA	1. PA	1. Abrasion 2. Bruises 3. Fractures 4. Laceration 5. Skin tear
Wiglesworth 2009 USA	1. PA	1. Bruises
Ziminski 2013 USA	1. PA	1. Bruises
Case report studies		
Chang 2013 USA	1. PA	1. Abrasions 2. Bruises 3. Defensive injury
Danesh 2015 USA	1. PA 2. N 3. SA	1. Bruises 2. Burns 3. Contusions 4. Defensive injury 5. Signs of nutritional deficiency
Gibbs 2014 USA	1. PA 2. SN 3. SA	1. Abrasion 2. Blunt trauma 3. Bruises 4. Burn 5. Hematoma 6. Laceration 7. Moisture-associated.skin damage 8. Poor oral dentition 9. Pressure sore/ulcer (decubitus, pressure) 10. Untreated skin cancer
Palmer 2013 USA	1. PA 2. SN 3. SA	1. Bruises 2. Ligature marks
Rohringer 2020 Canada	1. PA	1. Contusion 2. Hematoma 3. Fracture 4. Soft tissue swelling
Russo 2019 Italy	1. PA	1. Contusion 2. Fractures
Speck 2014 USA	1. SA	1. Petechiae
Young 2014 USA	1. PA 2. N	1. Bruises 2. Dislocation 3. Fractures 4. Ulcers
Wong 2017 USA	1. EA	1. Fractures 2. Bruises 3. Hematoma 4. Hemorrhage 5. Ecchymosis

*EA* elder abuse, *PA* physical abuse, *SA* sexual abuse, *N* neglect, *SN* self-neglect, *PsA* psychological abuse, *FA* financial abuse

**Table 8:** Results descriptive and case report studies: characteristics of physical signs and anatomic location

1st author Year Country	Characteristics	Anatomic location skull/brain Maxillofacial/dental/neck	Chest/abdomen/back	Extremities (upper/lower)	Pelvis/gluteal	Extra-genital (sexual abuse)	Miscellaneous
Descriptive studies							
Abath 2010 Brazil		1. Face: 13.7% of victims 2. Skull/neck:6.0% of victims	1. Chest/abdomen: 5.7% of victims	1. Upper limbs: 27.4% of victims 2. Lower limb(s)/ pelvic girdle: 6.8% of victims	1. Lower limb(s)/ pelvic girdle: 6.8% of victims		1. Injury more than one part of the body: 40.4% of victims
Burgess 2005 USA	1. Thumb/finger marks				1. Vaginal trauma: 46.2% of victims 2. Bruising labia minora: 37.8% of victims 3. Bruising of posterior fourchette:37.2% of victims 4. Bruising labia majora: 31.1% of victims other areas includedclitoris, fossa navicularis, vestibule, hymen, cervix, perineum, anus, rectum	1. Injury head: 38% of victims 2. Injuries arms: 31%of victims 3. Injuries legs: 24% of victims 4. Injuries chest: 22% of victims 5. Injuries abdomen: 13% of victims 6. Injuries other locations: 20% of victims	
Cham 2000 Singapore		1. Injuries maxillo-facial/head	1. Bruises chest 2. Contusions (sexual)	1. Fracture radius/ulna			
Kavak 2019 Turkey		1. Fractures head and neck: 30.4% of victims (mostly temporal, nasal and maxilla-orbita fracture) 2. Soft tissue lesions head and neck: 36% of victims	1. Fractures chest: 30.4% of victims (mostly multiple fractures of costae and located in the posterior segment) 2. Fractures lumbar /pelvic region: 4.3% of victims	1. Fractures upper extremities: 37% of victims (mostly humerus and ulna) 2. Fractures lower extremities: 26.1% of victims (mostly tibia and femur) 3. Soft tissue lesions of upper (32%); lower (40%) extremities 4. Long bone fractures: located in distal end of bone and diaphyseal bone segment in 56.9% and 53.8% of the cases, respectively 5. 77.2% of the bone fractures were non-displaced fractures and 12% of victims had a concurrent joint dislocation			1. Lesions were often on the left side of the body (54.3%) 2. Old fractures: 19.6% of victims
Rosen 2016 USA		1. Injuries on head, face and neck, notably, fractures and bruising maxillo-facial/dental/neck 2. Bruising to eye/orbit 3. Subdural hematoma and corneal abrasion 4. Lacerations to skull/brain	1. Bruises on breast 2. Fracture cervical spine 3. Fractures ribs 4. Lacerations to torso	1. Injuries and bruising/ dislocations on the upper (45% of visits)/lower extremities (32% of visits) 2. Fractures tibia/fibula/hip/femur 3. Lacerations to lower extremity	1. Fracture pelvis		
Rosen 2020 USA		1. Maxillofacial/ dental/neck without injuries to the upper or lower extremities; (more likely injuries left cheek and zygoma, or on neck of ear then in patients with unintentional injuries)	Injuries chest/back/abdomen	Upper extremity > lower extremity	Injuries to pelvis/ buttocks		Physical abuse victims were significantly more likely to have bruising and injuries on the maxillofacial, dental, or neck region; Abuse victims were less likely to have fractures or injuries on the lower extremities; Injuries to the head and neck without injury to other parts of the body were much more common in abuse victims Differences that were not significant between case patients and controls: 1. Injuries to the ulnar and posterior aspect of the forearm on either or both sides and to the left ulnar and posterior aspect of the forearm; 2. Injuries skull/brain
Wiglesworth 2009 USA	1. Size bruises > 5 cm (longest dimension), no bruising of 1 cm or less	1. Head (predominant on face) and neck: 20.8% of victims (*p* = 0.006)	1. Posterior torso: 14.6% of victims (*p* = 0.02)	1. Lateral aspect right arm: 25% of victims (*p* = 0.008)	1. Burn injuries on the back and buttocks from scalding water		1. Physically abused older adults knew more often the cause of their bruises (43 (89.6%) vs 16 (23.5%) of the comparison group
Ziminski 2013 USA	ND	1. Head and neck: 14.9% of victims 2. Victims who reported being punched or hit were signify-cantly more likely to have bruises on head and neck (*p* = 0.001) and right lateral upper arm (*p* = 0.027) 3. Persons who reported being beaten up were significantly more likely to report bruises on head and neck (*p* = 0.001)	1. Posterior torso: 10.4% of victims	1. Lateral/anterior arms: 34.3% of victims 2. Persons who reported being grabbed were significantly more likely to have lateral/anterior arm bruises (left anterior upper *p* = 0.003/lower arm *p* = 0.016)			1. Victims who reported being choked were significantly more likely to have bruises on lumbar region (*p* = 0.007), head and nek (*p* = 0.039) and left anterior upper arm (*p* = 0.004)
Case report studies							
Chang 2013 USA	1. Superficial abrasions and dermal hemorrhage: beaten narrow object 2. Stab wounds		1. Truncal injuries through stab wounds 2. Chest with superficial abrasions and dermal hemorrhage	1. Injury on the dorsal surface of hand (defensive injury)			
Danesh 2015 USA	1. Casal necklace due to vit B3 deficiency 2. Stocking distribution injury			1. Injury on the back of right hand (defensive injury) 2. Burn injury in stocking distribution at the extremities	Perianal contusions after sexual abuse		
Gibbs 2014 USA	1. Distinct bruising pattern from lying on bird seed on a hard floor 2. Pattern bruising from a ligature 3. Bruising in a tramline fashion 4. Burn from a curling iron	1. Bruising of the ear, called boxer ear 2. Poor oral dentition 3. Black eyes	1. Atypical bruising of the chest in a case of substantiated abuse 2. Bruising across the breast and upper arm from blunt trauma	1. Stage II heel ulcer	1. Moisture-associated skin damage and ulcers in the sacrum, buttocks, and thighs 2. Stage I and II ulcers on the buttocks and stage II–III on the lower back 3. Stage 3 sacral ulcer/Sacral decubitus ulcer		1. Case report author: male end stage dementia with sepsis from stage 4 sacral ulcer due to neglect 2. Blood tracking inferior to eye. Point of impact is seen as yellow bruising lateral to the eye 3. Untreated skin cancer in a case of neglect of an older man with dementia
Palmer 2013 USA	1. Fingertip-patterned bruising 2. Patterns injury suggestive implement use	1. Bruising medial aspect thigh 2. Bruising on ear		1. Ligature mark due to restraint on leg			
Rohringer 2020 Canada		1. Subcutaneous hematoma over the midline of the frontal bone 2. Soft tissue hematoma over the right frontal bone 3. Soft tissue swelling over the left orbit, fracture of the medial wall of the left orbit, and comminuted nasal bone fracture 4. Subcutaneous hematoma over the left side of the neck 5. Asymmetric left mandibular and parotid soft tissue swelling	1. Central cord contusion 2. Bilateral healed rib fractures	1. Left humeral neck fracture			
Russo 2019 Italy			1. Bruising on the posterior torso correlated to posterior rib fractures 2. Pulmonary contusion 3. Fracture at the middle third of the left clavicle and multiple ipsilateral rib fractures	1. Fracture diaphyseal part of the right humerus			
Speck 2014 USA					1. Punctate petechia on vestibular and vaginal tissues		
Young 2014 USA		1. Fractures of head 2. Bruises to the face	1. Fractures of cervical spine/trunk	1. Spiral fractures of the large bones of the limbs and fractures with a rotational component 2. Shoulder dislocation of the nondominant arm 3. Decubitus ulcers bilateral heels	Decubitus ulcer over the coccyx, right ischium		
Wong 2017 USA	1. Injuries in multiple stages of healing, 2. Multifocal fractures	1. Injuries maxillofacial region 2. Bilateral periorbital bruising, multiple ecchymoses over body and face 3. Bilateral nasal bone fractures 4. Left frontal scalp hematoma 5. Prior sub-arachnoid hemorrhage	1. Multifocal fractures of the bilateral ribs or specifically right posterior ribs	1. Distal ulnar diaphyseal fracture/chronic fracture deformity of the distal ulnar and distal radial diaphysis 2. Injuries upper extremities 3. Transverse fracture through the proximal humeral metadiaphysis 4. Age indeterminate fracture deformity of the right inferior pubic ramus 5. Acute fractures of the right clavicle	1. Acute fractures of the pelvis		Injuries inconsistent with reported mechanism

#### Types of physical signs

The most commonly described physical signs in elder abuse were bruises [12, 13, 22, 23, 25–27, 29, 30, 35–37].

#### Characteristics of physical signs

Wiglesworth et al. [13] described that with regard to the size of physical signs, bruises related to physical elder abuse are often large, e.g., > 5 cm wide at its widest point. Other studies described that with regard to the shape of physical signs, bruises and injuries related to elder abuse can be body part marked, e.g., the presence of thumb and finger marks (fingertip bruising) or object marked, e.g., ligature bruising or tramline bruising due to beating with a narrow shaped object [12, 22, 35, 37]. Furthermore studies described that the distribution of physical signs in a stocking or glove distribution (e.g., due to immersion of the extremities in hot water), the presence of a cutaneous casal necklace (dermatitis around the neck due to vitamin B3 deficiency in case of neglect), and injuries in multiple stages of healing or multifocal fractures to be caused by elder abuse [30, 36]. No description of characteristics of physical signs were given in six out of eight descriptive studies [21, 23–27], two out of nine case report studies (primary case report studies) [28, 29], and two out of six mixed methods studies [38, 39].

#### Anatomic location of physical signs

Anatomic locations of physical signs in elder abuse were described to be predominantly on the head, face/maxillofacial area (including eyes, ears and dental area), neck, upper extremities and torso (especially posterior). Other anatomic locations mentioned to be associated with elder abuse included the lower extremities, abdomen, lumbar area and gluteal/genital/rectal area, the latter location often mentioned as being associated with the presence of sexual elder abuse or neglect (e.g., decubitus ulcers) [12, 13, 21–27, 29, 30, 35–39].

Some studies described physical signs due to elder abuse to be specifically located on the left respectively right side of the body [13, 24, 26, 27, 30, 38, 39]. Furthermore Rosen et al. [26] described that physical abuse victims were more likely to have visible injuries in the maxillofacial, dental or neck area without the presence of injuries to the upper or lower extremities. Also, certain anatomic locations of bruises were described to be related to the mechanism of injury. The odds that a person had head and neck bruises were greater in case they were choked, punched and beaten up than in persons who did not report being choked, punched and beaten up. The odds of having bruises on the lateral/anterior arm were greater when persons reported to be grabbed compared to persons who did not report being grabbed [27]. Physical signs related to sexual elder abuse were mostly located in vestibular and vaginal tissues (petechiae), the labia minora and majora (bruising), posterior fourchette (bruising) and the perianal area (contusions). Victims of sexual elder abuse were furthermore described to have a significant amount of injury located at non-genital parts of their body, especially to their head and arms and on the medial aspect of the thigh [12, 22, 36]. Physical signs of sexual elder abuse in males were not found. In the article of Speck et al. 2014, only two cases of sexual abuse in males were described. Only one case was defined as sexual elder abuse, but in this case signs of physical injury were lacking [28]. Physical signs related to neglect were described as cutaneous lesions due to vitamin deficiency, poor oral dentition, physical signs on the surface of the skin due to untreated skin cancer or moisture, decubitus ulcers in the sacrum, buttocks, thighs and stage I–III decubitus ulcers on heels [36, 37].

### Additional outcomes (see Table 9)

**Table 9:** Results reviews and books: Types and characteristics of physical signs and anatomic location

1st author Year Country	Types EA	Types physical signs	Summary characteristics: physical signs	Summary anatomic location physical signs	Miscellaneous
Reviews					
Brown 2004 Country not described	1. PA 2. SA	1. Abrasions 2. Bruises 3. Fractures 4. Lacerations 5. Contusion 6. Petechiae /ecchymosis 7. Bleeding	1. Fingertip bruising 2. Punch bruising 3. Strangulation signs	1. Fingertip bruising from restraint on neck, arms, and/or legs 2. Punch bruising on face, breasts, chest, abdomen, and extremities 3. Chest wall injuries: rib fractures 4. Fingertip bruising from sexual abuse on inner/outside thighs 5. Genital injury (bruising or bleeding) 6. Fractures extremities (defense or fall) 7. Cervical spine injuries 8. Location signs in sexual assault (% victims): chest wall injury (22%), head injury (38%), abdominal injury (15%), injury arms (30%), bruising on legs (> 20%), vaginal trauma (45%), anal trauma (17%), oral penetration (13%)	1. Document physical injuries and signs using acronym “TEARS": Tears or lacerations and/or tenderness; Ecchymosis; Abrasions; Redness; Swelling 2. Skin of elderly has a slower healing rate
Chang 2013 USA	1. PA 2. N 3. SA	1. Abrasions 2. Alopecia (traumatic) 3. Bleeding 4. Burns 5. Bruises 6. Cutaneous signs nutritional deficiency 7. Dermatitis 8. Dislocations 8. Erosions 9. Erythema 10. Fractures 11. Lacerations 12. Poor hair/nail care 13. Purpura or petechiae 14. Scars 15. Signs sexual transmitted disease 16. Ulcers (pressure)	1. Patterned shape or distribution 2. Various stages healing 3. Bilateral or parallel injuries 4. Irregular patches of alopecia	1. Cutaneous manifestations female sexual abuse can involve extragenital and genital sites: (% victims) -Genital: vagina (46%), labia minora (38%), posterior fourchette (37%), and labia majora (31%) -Extragenital: head (38% of all cases) and arms (31%), oropharynx and anorectal areas 2. Unexplained sexual transmitted diseases (genital or skin or oral)	1. Sexual abuse signs: torn or stained underwear, difficulty walking or sitting without clear reason, or pain or bleeding from the genital area 2. Nutritional deficiencies from elder neglect can lead to a variety of skin manifestations
Clarysse 2018 Belgium	1. PA 2. N 3. SA	1. Abrasions 2. Alopecia (traumatic) 3. Burns 4. Bruises 5. Cutaneous signs nutritional deficiency/malnutrition 6. Dehydration 7. Fractures 8. Lacerations 9. Multiple ulcers (decubitus) 10. Purpura 11. Signs sexual transmitted disease	1. Patterned shape or distribution 2. Different healing stages of lesions, e.g., healing by secondary intention 3. Parallel injuries 4. “Tram lines” 5. Irregular patches of alopecia 6. Deep- and/or foul-smelling necrotic ulcers 7. Stocking, glove distribution 8. Cigarette burns	1. Laceration located around the eye, nose, or mouth 2. Spiral fracture of long bones or in other sites than wrists, vertebrae, and hips when free from alcohol/substance abuse 3. Fractures of the zygomatic ark, mandible, and maxilla 4. Ligature marks around wrists and ankles 5. Alopecia outside the vertex and frontotemporal area, hemorrhages or hematomas present at the site of hairloss 6. Glossitis, heilitis and/or dermatitis due to nutritional deficiencies 7. Fingertip-patterned abrasions and bruises located on the inner thighs of the victim 8. Oral erosive ulcerations, bruises of the uvula, or the palate in sexual abuse	1. The color of bruising is not reliable for age determination 2. Sudden pain or bleeding of the anogenital area and impaired walking of elderly
Collins 2006 USA	1. PA 2. N 3. SA	1. Abrasions 2. Alopecia (traumatic) 3. Asphyxia signs 4. Bite marks 5. Burns 6. Contusions 7. Dehydration signs 7. Ecchymoses 8. Fractures 10. Poor hygiene signs 11. Malnutrition signs 12. Ulcers (decubitus) non-lumbar/non-sacral areas	1. Contusions multiple and clustered 2. Unusual alopecia pattern 3. Sexual abuse: injuries secondary to restraints	1. Contusions: Inner arms/thighs, palms/soles, scalp, ear (pinna), mastoid area, buttocks, on various planes of the body 2. Abrasions: axillary (restraints) wrist and ankles (ligatures) 3. Nasal bridge and temple injury (eyeglasses), periorbital ecchymoses, oral injury 4. Decubitus ulcers in non-lumbar/sacral area, fracture not hip/humerus/vertebra 5. Non-genital trauma in sexual abuse: hard and soft palate trauma	1. Untreated fracture/decubitus ulcers 2. Non-genital trauma in sexual abuse: signs of asphyxia
Danesh 2015 USA	1. PA 2. N 3. SA	1. Alopecia (traumatic) 2. Bruises 3. Burns (immersion) 4. Contusions 5. Dehydration 6. Dermatitis 7. Lacerations 8. Malnutrition signs 9. Purpura 10. Ulcers (decubitus) 11. Signs sexual transmitted disease 12. Poor general and/or oral hygiene	1. Size > 5 cm 2. Resembles implement used 3. Foul-smelling decubitus ulcers 4. Stocking/glove distribution (immersion burns)	1. Injury located on face, right side of arm or torso 2. Decubitus ulcer outside of sacral or lumbar region 3. Extragenital manifestations of abuse 4. Genital bleeding	1. Torn or stained underwear 2. Difficulty walking without clear reason 3. Pain in genital area
Gibbs 2014 USA	1. PA 2. SN/N 3. SA	1. Abrasions 2. Avulsions 3. Bite marks 4. Bruises 5. Burns 6. Fractures 7. Poor hygiene signs 8. Rashes 9. Skin tears 10. Moisture-associated skin damage 11. Ulcers (pressure)	1. Bruising in older adults does not always follow standard color progression; one cannot reliably predict the age of a bruise by its color 2. Bruise size > 5 cm 3. Multiple bruises of varying ages 4. Incised wounds caused by a sharp-edged object 5. Scalds from hot water with (struggle) or without (immobile pt) presence splash marks 6. Bilateral, or stocking and glove injuries, skin sparing with surrounding burn area or hot objects leaving pattern	1. Subgaleal hematoma after traumatic hair pulling 2. Tracking in perineum after genital trauma 3. No accidental bruises are found on the neck, ears, genitalia, buttocks, or soles 4. Bruising on lateral right arm, and to the head and neck 5. Injuries head and torso 6. Injuries upper extremities and maxillofacial regions, torso 7. Bruising from sexual abuse located on labia majora,labia minora, or posterior fourchette 8. Defensive stab wounds on the inner(volar) side of the wrist or forearm 9. Lacerations and abrasions in the genital area 10. Head, neck, and face are the most common areas of injury 11. Physical signs strangulation: patterned abrasions or contusions of the anterior neck; hand marks may be the victim’s 12. Physical signs strangulation: petechiae on the neck, head, face, forehead, eyes, ears, conjunctivae, and buccal mucosa 13. Signs of basilar skull fracture (raccoon eyes/battle signs)	1. Hoarseness in strangulation cases 2. Signs of strangulation: difficulty swallowing, dyspnea, and stridor 3. Signs of strangulation: assuming a sniffing position to assist with breathing 4. Injuries from falls: cranio-maxillofacial injury, brain trauma, upper and lower extremity injury, and thoracic injury 5. Case report author: male end stage dementia with sepsis from stage 4 sacral ulcer due to neglect
Murphy 2013 Canada	1. PA 2. SA	1. Abrasions 2. Bruises 3. Burns 4. Contusions 5. Fractures 6. Hemorrhages (subdural)	1. Mostly large bruising	1. Injury to the upper extremity 2. Maxillofacial and upper extremity injuries: upper extremity injuries were mostly categorized as shoulder and arm nonspecific injury; maxillofacial and head and the neck injuries were mostly located periocular and eyelid region 3. Subdural hemorrhages, subcutaneous hemorrhages (head and neck region) 4. Preponderance of injury to the head and torso 5. Bruises on the face, posterior torso, and lateral right arm 6. Blunt musculoskeletal trauma 7. Injuries to posterior torso and lower extremity, inner thigh, or dorsal or plantar aspect foot Of the 839 injuries in this review, the distribution by anatomic region was as follows: upper extremity (43.98%), maxillofacial and neck (22.88%), skull and brain(12.28%), lower extremity (10.61%), and torso (10.25%)	
Palmer 2013 USA	1. PA 2. SN/N 3. SA	1. Abrasions 2. Burns 3. Bruises 4. Lacerations 5. Decubitus ulcers 6. Traumatic alopecia 7. Purpura 8. Signs sexual transmitted disease 9. Poor hygiene signs 10.malnutrition	1. Patterns of injury and patterned injury 2. Bruising: most seen lesions 3. Bruises larger than 5 cm 4. Punches: shape of fist with area of central clearing 5. Color of a bruise not indicative of age 6. Patterns burns elder abuse are similar to child abuse: -immersion burn in a stocking and glove distribution -injuries resemble implement 7. Abrasions with patterns parallel to the force that inflicted the injury 8. Single or multiple patchy areas of alopecia, with or without hair breakage, outside normal pattern, especially if with hemorrhage or hematoma	1. Bruises located on the face, side of right arm, or back of torso 2. Bruising by punch on the face, breast, chest, abdomen, or extremities 3. Laceration or abrasions to the eye, nose, or mouth 4. Lacerations by blunt force most commonly were skin is closely opposed by bone 5. Abrasions or scars around the ankle, wrist, or axillae from restraints 6. Bruising of the labia majora, labia minora, or posterior fourchette 7. Signs genital trauma like: erythema, lacerations, abrasions, and genital pain or tenderness and sexual transmitted diseases 8. Fingertip-patterned bruising, and abrasions on the inner thighs especially in combination with other signs of elder abuse 9. Oral injury such as contusion or lacerations of the inner lips, buccal mucosa, or edentulous ridges indicative sexual abuse or force feeding 10. Signs neglect: dry mucous membranes, sunken eyes, or decreased skin turgor in dehydration; untreated decubitus ulcers; poor hygiene	
Pearsall 2005 USA	1. PA 2. N 3. SA	1. Abrasions 2. Bruises 3. Burns 4. Dehydration 5. Excoriations 6. Fractures 7. Lacerations 8. Ulcera (decubitus) 9. Poor hygiene signs 10. Signs sexual transmitted disease	1. Bruise with the shape of knuckles or fingers; parallel discoloration marks a linear cylindrical object 2. Bruise with central clearing from fist punch	1. Fingertip bruising from restraint on neck. Arms, and legs 2. Bruises from punches on breast. Chest. Abdomen, and extremities 3. Bruising to the inner thigh in sexual abuse 4. Reddened, ecchymosed, itching or painful genital area in sexual abuse 5. Suggestive sexual abuse: oral venereal lesions, bruising of the uvula or palate, new diagnosis sexual transmitted disease	1. Signs of difficulty sitting or walking, bloody or stained undergarment in sexual abuse
Rohringer 2020 Canada	1. PA	1. Bruises 2. Dislocation 3. Fractures 4. Hematomas		1. study 1: percentage injuries to: upper extremities (43.98%), maxillofacial, dental and neck region (22.88%), the skull and brain (12.28%), the lower extremities (10.61%) and the torso (10.25%) 2. Study 2: percentage injuries to: upper extremities (45%), followed by head and neck injuries (42%), and lower extremities (32%) 3. Injured areas: head and neck, followed by chest, breasts and abdomen 4. Internal injury pelvis, bladder and ureter 5. Fall-related injuries in association with abuse: bruises on the breast, internal injuries, and upper extremity dislocations 6. Anterior sternal dislocations, ectopia lentis and depressed skull fractures 7. Injuries to head and torso 8. Visible bruising on upper extremities 9. Bruising location most common: lateral/anterior arms (34.3%), followed by the head and neck (14.9%) and the posterior torso 10. Odds lateral/anterior arm bruises 8 × times greater when grabbed; odds head/neck bruises greater when choked or beaten 11. Posterior torso bruising and ulnar forearm bruising 12. Injuries to the neck and left face 13. Multiple (misaligned) healed fractures 14. Injuries upper extremities and maxillofacial region 15. Bruising on the posterior torso in association with posterior rib fractures, and bruising on the ulnar forearm in association with distal ulnar diaphysis fractures 16. Anterior sternoclavicular dislocations 17. Upper rib fractures	
Russo 2019 Italy	1. PA	1. Bruises 2. Fractures	1. Restraint marks	1. Bruising of the ulnar forearm from defense measures 2. Fracture of the distal ulnar diaphysis 3. Contusions and abrasions to the axilla and inner aspects of the arms 4. Bruising on the lateral aspect of the arm 5. Injuries to posterior torso and lower extremity, inner thigh, or dorsal or plantar aspect of the foot 6. Injuries in upper extremities 7. Injuries to the brain, head, and neck 8. Injuries in multiple stages of healing, particularly in maxillofacial region and upper extremities; injury patterns uncommon in accidental injury, such as ulnar diaphysis fracture	1. Injuries inconsistent with reported mechanism
Books					
Baccino 2020 France	1. PA 2. N	1. Burns 2. Cutaneous ecchymosis 3. Bruises 4. Dehydration 5. Hematomas 6. Scars 7. (poor) (oral) hygiene signs		1. Bruising on the back and lateral aspects of forearms and wrists 2. Trauma to temporal area, eyes and nose, breast, inner aspect of arm skin 3. Injuries to upper limbs (43.98%) > maxillofacial region, teeth and neck (22.88%) > skull and brain (12.28%) > lower limbs (10.61%) > trunk (10.25%) 4. Subdural hematoma, possible shaken granny syndrome exists	1. An injury, which does not appear to match with the proposed mechanism 2. Skin lesions of different colors suggesting repeated trauma
Dyer 2003 USA	1. PA 2. N 3. SA	1. Abrasions 2. Bruises 3. Burns 4. Dehydration 5. Fractures 6. Lacerations 7. Malnutrition signs 8. (poor) hygiene signs 9. Signs sexual transmitted disease 10. Ulcera (decubitus)	1. Bruises can retain shape of knuckles or fingers; parallel marks, called tramline bruising, indicate injury from stick 2. Color of bruise unhelpfull for dating, but reddish blue, blue or purplish bruises seem more recent as opposed to bluish green, greenish yellow, and brown bruises 3. Multiple bruises in various stages of healing 4. Foul-smelling or necrotic ulcer 5. Large skin tears or excessive scarring from more serious lacerations without adequate explanation 6. Circular bruising, especially bilaterally from forcibly lifting 7. Parallel lines caused by impact by a rounded or cylindrical object or an unusual pattern	1. Injury to face and neck, the chest wall, the abdomen, and the buttocks 2. Intentional injury to head and internal injuries 3. Bruising on the palms and soles 4. Fractures of the head, spine, and trunk are more likely to be assault injuries than limb fractures, sprains or strains, or musculoskeletal injuries 5. Scars or wrist wounds of decubitus due to restraints 6. Oral venereal lesions 7. Bruising of the uvula and bruising of the palate and the junction of the hard palate may indicate forced oral copulation 8. Bruising, inflammation, tenderness, abrasions, or trauma of anogenital area 9. Extragenital signs sexual abuse: bruising abdomen 10. Injuries suggestive of defensive maneuvering, such as on the back of the arms and hands, and injuries related to grasping, squeezing, or forcible restraint	

*EA* elder abuse, *PA* physical abuse, *SA* sexual abuse, *N* neglect, *S N* self-neglect, *PsA* psychological abuse, *FA* financial abuse, *ND* not described

Additional outcomes of elder abuse were diverse and involved wounds and unexplainable injuries, combinations of injuries, mechanism of injuries, sexual elder abuse and neglect in victims. Additional characteristics of physical signs were deep and/or foul-smelling necrotic aspects of ulcers, bilateral or parallel and irregular injuries, multiple and clustered injuries, circular bruising, splash marks from hot water and traumatic/irregular patches of alopecia. Although the color of bruises was stated not reliable for the dating of bruises, bruises with differing colors may point at recurrent abuse [12, 32, 33, 35, 37, 41]. Anatomic locations of specific injuries in elder abuse were: a basilar skull fracture due to elder abuse (raccoon sign or periorbital ecchymosis) and bruising over the mastoid process (battle sign). In (attempted) strangulation, the following physical signs were described: abrasions on anterior neck and petechiae on neck, head, face, eyes, ears, conjunctivae and buccal mucosa [37]. Additionally to the anatomic locations of physical signs, it was mentioned that bruising to the ulnar side of the forearms of victims of elder abuse was often combined with the presence of a fracture of the distal ulnar diaphysis, and that bruising to the posterior torso was often combined with rib fractures [38]. Finally, injuries to palms and dorsal or plantar soles of the feet were also mentioned as physical signs of elder abuse. In victims of sexual elder abuse, additional anatomic locations of physical signs were unexplained sexually transmitted diseases (located on genital area or skin or oral area), pain or bleeding from the genital area, bruising to the uvula or the palate and lacerations to inner lips and buccal mucosa [32, 33, 35]. In case of neglect, dry mucous membranes, sunken eyes or decreased skin turgor in dehydration and poor general hygiene were described [12, 37].

## Discussion

The most commonly described physical signs in elder abuse were bruises. Characteristics of physical signs could be categorized into size, shape and distribution. Physical signs were anatomically predominantly located on the head, face/maxillofacial area (including eyes, ears and dental area), neck, upper extremities and torso (especially posterior). Physical signs related to sexual elder abuse were mostly located in vestibular and vaginal tissues, the labia minora and majora, posterior fourchette and the perianal area. Victims of sexual elder abuse were furthermore described to have a significant amount of injury located at non-genital parts of their body, especially on their head and arms and the medial aspect of the thigh. Unfortunately, with regard to the characteristics and anatomical location of physical signs in sexual elder abuse in older males, information was absent.

This is the first systematic review on the state-of-the-art knowledge on physical signs in elder abuse where a quality analysis of observational studies was performed and additional findings of other designs were included. Furthermore, physical signs were described and classified along the lines of the taxonomy instrument for visible intentional and unintentional acute injuries based on the study by Rosen et al. [20]. By identifying the types, characteristics and anatomic location of physical signs in elder abuse, this review contributes to the awareness and recognition of elder abuse by clinical geriatricians and other healthcare professionals. Detecting specific injury patterns suggestive of elder abuse can aid healthcare professionals in their physical examination and strengthen the need for a head to toe examination. The use of a taxonomy instrument for a structured and uniform description of characteristics and location of physical signs in elder abuse can help healthcare professionals to systematically assess physical signs, especially in situations where it is not easy to discriminate from signs of other underlying diseases. To move forward on the road to early detection and awareness of physical and other signs of elder abuse, it is necessary to invest in education. In contrast to pediatricians educated in the recognition of and care for child abuse victims, education on the recognition of physical signs in elder abuse (and other signs of elder abuse, e.g., in financial and physiological abuse) is not yet common for clinical geriatricians and other healthcare professionals (such as nurses) in clinical care. Also, the sense of ownership and commitment regarding the recognition and care of elder abuse victims is not yet self-evident in geriatric healthcare professionals. The authors of this review strongly recommend education on this topic, not only for clinical geriatricians but for all other healthcare professionals with a caseload of older patients. Furthermore, to effectively deal with elder abuse, a systematic screening for a timely identification of signals, as well as a systematic approach in case elder abuse is (suspected to be) present, is necessary. With regard to an effective screening on elder abuse, no single tool has yet been found appropriate [42, 43]. In absence of an appropriate validated tool for signalling elder abuse, the Dutch guideline on (suspected) elder abuse [17] recommends that healthcare professionals working in the hospital setting should be aware of an internal sense of alarm with regard to the (possible) presence of elder abuse, by asking themselves a “gut feeling” question in 70 + individuals that visit the hospital setting. Unfortunately, effective screening on elder abuse is not enough. It is equally important to have an adequate approach and follow-up process in each hospital or nursing home, when cases of elder abuse are suspected and/or present. Since 1 July 2013, it is mandatory for professionals in the Netherlands to follow a mandatory reporting code in case of (suspected) domestic violence and child abuse (source: Government of the Netherlands (https://www.government.nl/topics/domestic-violence/domestic-violence-and-child-abuse-protocol). In the Netherlands, elder abuse is categorized as a form of domestic violence and thus in case of elder abuse the mandatory reporting code in case of (suspected) domestic violence and child abuse is followed. Cases of elder abuse as a result of abuse by healthcare professionals are primarily reported to the healthcare inspectorate.

The reporting code offers a five-step plan detailing the best course of action and helps healthcare professionals in and outside (clinical) geriatric care to decide whether or not to report the situation to the Adult Protective Services (APS). In addition, within each hospital or institutional setting caring for older persons, it would be advisable to appoint a case manager on domestic violence and elder abuse, to coordinate and guide compliance with the follow-up of the reporting code and to support and advise the healthcare professional in the recognition and care for victims of elder abuse. The installation of an additional Multidisciplinary Elder Abuse Team (MEAT), where cases of elder abuse victims are (anonymously) discussed and course of action is evaluated, could further enhance a multidisciplinary approach to elder abuse investigation. Participants should at minimum include clinical geriatric and emergency medicine experts (nurses and physicians), a case manager on domestic violence and elder abuse, a social worker and a representative of the regional Adult Protective Services. With regard to the recognition of physical signs in elder abuse, it would be advisory not only to include forensic expertise from a forensic physician or a forensic trained nurse in the multidisciplinary elder abuse team, but also to incorporate them as a consultant in the acute setting. They can help recognize and safeguard forensic evidence during the assessment process. Finally, thorough reporting and transfer to healthcare professionals during discharge/care transition is essential in this process.

A key limitation of this systematic review is that the primary data studies had moderate methodological quality and included only a limited number of studies with a (matched) control group. Furthermore, the narrative reviews mainly summarized the included observational descriptive studies. However, with this review, a contribution and an incentive to achieve higher methodological research quality in the field of elder abuse have been made, as pitfalls in existing knowledge on physical signs of elder abuse have been identified. More research regarding for example pathognomic injuries in elder abuse could eventually provide healthcare professionals with (more) practical knowledge on adequate and timely recognition of physical signs in elder abuse.

## Conclusions

- The most commonly described physical signs in elder abuse are bruises.
- Older persons are more likely to have physical signs of elder abuse located on the head, face/maxillofacial area (including eyes, ears and dental area), neck, upper extremities and torso (especially posterior).
- Physical signs related to sexual elder abuse are mostly located in the genital and perianal area and are often accompanied by a significant amount of injury to non-genital parts of their body, especially to the area of the head, arms and the medial aspect of the thigh.
- The characteristics and anatomical location of physical signs in sexual elder abuse in males needs to be explored in future research.
- Knowledge regarding the most common types, characteristics and anatomic location of physical signs in elder abuse is useful to increase the awareness and recognition of elder abuse by clinical geriatricians and other healthcare professionals.
- There is a need for education on physical signs in elder abuse; furthermore, this topic should be included in clinical curricula at different levels (i.e., pre- and post-qualification): not only in bachelor and master programs for professionals such as clinical geriatricians and emergency physicians, but also for nursing and other healthcare professionals.

## Supplementary Information

Below is the link to the electronic supplementary material.

Supplementary file1 (DOCX 17 KB)

## Data Availability

In line with the nature of the systematic review, original research papers included in the article were provided with references.

## Code availability

Not applicable for the research design.
