# Supplementary material for: Types, characteristics and anatomic location of physical signs in elder abuse: a systematic review: Awareness and recognition of injury patterns
Source: Eur Geriatr Med. 2021 Sep 13;13(1):53–85. doi: 10.1007/s41999-021-00550-z (PMC8860961; doi:10.1007/s41999-021-00550-z)
Supplement: Supplementary file 1 — Supplementary file1 (DOCX 17 KB) [file 41999_2021_550_MOESM1_ESM.docx]

**Full search strategies per database**

**Medline 13-7-2020**

1 elder abuse/ or physical abuse/ or violence/ or domestic violence/ or torture/ or Restraint, Physical/ or homicide/ or sex offenses/ or rape/

2 ((physical or sexual or elder*) adj2 (harm or abus*)).ti,ab,kf.

3 (neglect* or ill-treat* or maltreat* or mistreat* assault* or crime* or violen* or aggression or coerc* or extort*).ti,ab,kf.

4 1 or 2 or 3

5 exp *Aged/ or exp *geriatrics/ or exp Geriatric Assessment/ or exp *Alzheimer Disease/ or exp Homes for the Aged/ or exp Nursing Homes/

6 (community-dwelling or alzheimer* or frail* or ageing or aging or dement* or psychogeriatric* or geriatric* or elder* or "cognitive impairment*" or old-age* or pensioner* or retire*).ti,ab,kf. or (aged or old or older).ti.

7 5 OR 6

8 4 AND 7

9 (Screen* or detect* or identif* or predict*).ti,ab,kf.

10 ((suspic* or sign* or symptom*) adj3 (abuse or mistreatment or bruis*)).ti,ab,kf.

11 Physical Examination/

12 head-to-toe.ti,ab,kf.

13 Examination.ti,ab,kf.

14 forensic medicine/ or forensic pathology/ or forensic nursing/ OR exp Hemorrhage/

15 (forensic* or legal*).ti,ab,kf.

16 9 or 10 or 11 or 12 or 13 or 14 or 15

17 8 AND 16

18 limit 17 to yr="2005 -Current"

**Cochrane 13-7-2020**

#1 ((physical or sexual or elder*) NEAR/2 (harm or abus*)):ti,ab,kw

#2 (neglect* or ill-treat* or maltreat* or mistreat* assault* or crime* or violen* or aggression or coerc* or extort*):ti,ab,kw

#3 #1 or #2

#4 (community-dwelling or alzheimer* or frail* or ageing or aging or dement* or psychogeriatric* or geriatric* or elder* or "cognitive impairment*" or old-age* or pensioner* or retire*):ti,ab,kw

#5 (aged or old or older):ti

#6 #4 OR #5

#7 #3 AND #6

#8 (Screen* or detect* or identif* or predict*):ti,ab,kw

#9 ((suspic* or sign* or symptom*) NEAR/3 (abuse or mistreatment or bruis*)):ti,ab,kw

#10 head-to-toe:ti,ab,kw

#11 Examination:ti,ab,kw

#12 (forensic* or legal*):ti,ab,kw

#13 #8 OR #9 OR #10 OR #11 OR #12

#14 #7 AND #13

**Cinahl 13-7-2020**

S1 MH "Elder Abuse" OR MH "Violence" OR MH "Domestic Violence" OR MH "Patient Abuse" OR MH "Patient Assault" OR MH "Intimate Partner Violence" OR MH "Exposure to Violence" OR MH "Gender-Based Violence" OR MH "Torture" OR MH "Restraint, Physical" OR MH "Homicide" OR MH "Sexual Abuse" OR MH "Rape"

S2 TI ((physical or sexual or elder*) N2 (harm or abus*)) OR AB ((physical or sexual or elder*) N2 (harm or abus*)) OR SU ((physical or sexual or elder*) N2 (harm or abus*))

S3 TI (neglect* or ill-treat* or maltreat* or mistreat* assault* or crime* or violen* or aggression or coerc* or extort*) OR AB (neglect* or ill-treat* or maltreat* or mistreat* assault* or crime* or violen* or aggression or coerc* or extort*) OR SU (neglect* or ill-treat* or maltreat* or mistreat* assault* or crime* or violen* or aggression or coerc* or extort*)

S4 S1 or S2 or S3

S5 MM "Aged+" OR MH "Dental Care for Aged" OR MM "Geriatrics" OR MH "Geriatric Assessment+" OR MM "Alzheimer's Disease" OR MH "Nursing Home Patients" OR MH "Nursing Homes+"

S6 TI (community-dwelling or alzheimer* or frail* or ageing or aging or dement* or psychogeriatric* or geriatric* or elder* or "cognitive impairment*" or old-age* or pensioner* or retire*) OR AB (community-dwelling or alzheimer* or frail* or ageing or aging or dement* or psychogeriatric* or geriatric* or elder* or "cognitive impairment*" or old-age* or pensioner* or retire*) OR SU (community-dwelling or alzheimer* or frail* or ageing or aging or dement* or psychogeriatric* or geriatric* or elder* or "cognitive impairment*" or old-age* or pensioner* or retire*) OR TI (aged or old or older)

S7 S5 OR S6

S8 S4 AND S7

S9 TI (Screen* or detect* or identif* or predict*) OR AB (Screen* or detect* or identif* or predict*) OR SU (Screen* or detect* or identif* or predict*)

S10 TI ((suspic* or sign* or symptom*) N3 (abuse or mistreatment or bruis*)) OR AB ((suspic* or sign* or symptom*) N3 (abuse or mistreatment or bruis*)) OR SU ((suspic* or sign* or symptom*) N3 (abuse or mistreatment or bruis*))

S11 MH "Physical Examination" OR MH "Sexual Assault Examination" OR MH "Inspection Clinical" OR MH "Palpation" OR MH "Percussion"

S12 TI “head-to-toe” OR AB “head-to-toe” OR SU “head-to-toe”

S13 TI Examination OR AB Examination OR SU Examination

S14 MH "Forensic Medicine" OR MH "Forensic Pathology" OR MH "Forensic Nursing" OR MH "Sexual Assault Examination" OR MH "Hemorrhage+"

S15 TI (forensic* or legal*) OR AB (forensic* or legal*) OR SU (forensic* or legal*)

S16 S9 or S10 or S11 or S12 or S13 or S14 or S15

S17 S8 AND S16

S18 limit 17 to yr="2005 -Current"

**Embase 13-7-2020**

1 elder abuse/ or physical abuse/ or exp sexual abuse/ OR violence/ or assault/ or battering/ or exp domestic violence/ or exposure to violence/ or physical violence/ or exp sexual violence/ or torture/ OR homicide/

2 ((physical or sexual or elder*) adj2 (harm or abus*)).ti,ab,kw.

3 (neglect* or ill-treat* or maltreat* or mistreat* assault* or crime* or violen* or aggression or coerc* or extort*).ti,ab,kw.

4 1 or 2 or 3

5 exp *aged/ OR exp *geriatrics/ or Geriatric Assessment/ or *Alzheimer Disease/ or home for the aged/ OR nursing home/

6 (community-dwelling or alzheimer* or frail* or ageing or aging or dement* or psychogeriatric* or geriatric* or elder* or "cognitive impairment*" or old-age* or pensioner* or retire*).ti,ab,kw. or (aged or old or older).ti.

7 5 OR 6

8 4 AND 7

9 (Screen* or detect* or identif* or predict*).ti,ab,kw.

10 ((suspic* or sign* or symptom*) adj3 (abuse or mistreatment or bruis*)).ti,ab,kw.

11 physical examination/

12 head-to-toe.ti,ab,kw.

13 Examination.ti,ab,kw.

14 exp bleeding/ OR exp *injury/ OR exp forensic medicine/ or forensic nursing/

15 (forensic* or legal*).ti,ab,kw.

16 9 or 10 or 11 or 12 or 13 or 14 or 15

17 8 AND 16

18 limit 17 to yr="2005 -Current"

19 limit 18 to conference abstract status

20 18 NOT 19
